# Exosomes from tamoxifen-resistant breast cancer cells transmit drug resistance partly by delivering miR-9-5p

**DOI:** 10.1186/s12935-020-01659-0

**Published:** 2021-01-15

**Authors:** Jianhui Liu, Shaoliang Zhu, Wei Tang, Qinghua Huang, Yan Mei, Huawei Yang

**Affiliations:** 1 The First Department of Breast Surgery, Guangxi Medical University Cancer Hospital, Nanning, 530021 People’s Republic of China; 2 Department of Hepatobiliary Surgery, Guangxi Medical University Cancer Hospital, No.71, Hedi Road, Nanning, 530021 Guangxi People’s Republic of China

**Keywords:** Breast cancer, Drug resistance, Exosomes, MicroRNA-9-5p, ADIPOQ, Tamoxifen, MCF-7, MCF-7/TAM

## Abstract

**Background:**

Resistance to drug therapy is a major impediment for successful treatment of patients suffering from breast cancer (BC). Tamoxifen (TAM) is an extensively used therapeutic agent, which substantially reduces the risk of recurrence and associated mortality in BC. This study demonstrated that exosomal transfer of microRNA-9-5p (miR-9-5p) enhanced the resistance of MCF-7 cells to TAM.

**Methods:**

Initially, BC-related differentially expressed genes (DEGs) and their upstream regulatory miRNAs were identified. The TAM-resistant MCF-7 (MCF-7/TAM) cell line and the non-medicated sensitive MCF-7 cell line were formulated, followed by isolation of the exosomes. Next, the apoptosis rate of exosome-treated MCF-7 cells was determined after co-culture with TAM. The interaction between miR-9-5p and ADIPOQ was identified by a combination of bioinformatic analysis and luciferase activity assay. In order to validate the effect of miR-9-5p and ADIPOQ on TAM resistance in the MCF-7 cells in vitro and in vivo, miR-9-5p was delivered into the exosomes. ADIPOQ and miR-9-5p were identified as the BC-related DEG and upstream regulatory miRNA.

**Results:**

Exosomes derived from the MCF-7/TAM cells could increase the resistance of MCF-7 cells to TAM. Notably, miR-9-5p altered the sensitivity of BC cells to TAM. In addition, ADIPOQ was negatively regulated by miR-9-5p. Furthermore, MCF-7/TAM cell-derived miR-9-5p inhibited the apoptosis of MCF-7 cells, and promoted the cell resistance to TAM. In vivo experiments in nude mice ascertained that the tumor injected with exosomal miR-9-5p showed improved resistance to TAM.

**Conclusions:**

Exosomal transfer of miR-9-5p augmented the drug resistance of BC cells to TAM by down-regulating ADIPOQ, suggesting its functionality as a candidate molecular target for the management of BC.

## Background

Breast cancer (BC) is the most frequently occurring malignancy in females with a rising incidence worldwide [1, 2]. In China, BC is regarded the 6th leading cause of cancer-associated mortality [3]. The currently adopted treatment modalities for BC include surgical resection, chemotherapy, endocrine therapy, radiation therapy, and integrative multi-modal therapies [4, 5]. Tamoxifen (TAM) has been extensively used as a therapeutic agent for BC with a decreasing invasive potential capacity, although it has also been associated with adverse outcomes [6, 7]. However, TAM resistance is a frequent manifestation during BC management, which may be attributed to the high tumor levels of estrogen receptor [8].

Extracellular vesicles, including exosomes, have been examined in regard to anti-cancer investigations as modes of provision for anti-cancer drugs, as they may reduce resistance and entail fewer adverse effects than the free drugs [9]. Exosomes have been defined as homogeneously shaped vesicles, with a size of 40-00 nm in diameter [10]. While exosome-delivered molecules may hasten oncogenesis, aggressiveness, and drug resistance in BC, some exosomes can also transport anti-cancer drugs into BC cells, thereby minutely reducing resistance [11]. In the cancer microenvironment, the cancer cell-secreted exosomes and microRNAs (miRNAs) can be internalized by other cells, shuttled in the exosomes, and delivered to the recipient cells to mediate gene expression [12]. Accumulating evidence has ascertained the functionality of miRNAs as critical regulators of chief genes associated with drug resistance, and traditional therapies, moreover, in combination with miRNA-based treatment, it may be a promising option for the management of drug resistant BC [13].

In this study, we utilized microarray-based bioinformatic analysis to identify the differentially expressed gene ADIPOQ and its regulatory miRNA microRNA-9-5p (miR-9-5p) for subsequent experimentation. Previous bioinformatics prediction and functional assays have identified an association between the functional miRNA-mRNA networks with the invasiveness of BC cells [14]. An existing study elicited the clinicopathological role of miR-9 in BC metastasis, where it was highly expressed in the primary breast tumors from patients with advanced BC [15]. Additionally, miR-9-5p has been identified to facilitate the proliferation, migration, and invasion of non-small cell lung cancer cells by targeting and negatively regulating the TGFBR2 expression [16]. ADIPOQ, a gene encoding for adiponectin, is present on chromosome 3q27, and principally comprises of three exons and two introns [17]. Elevated ADIPOQ expression is associated with a superior survival rate of BC patients receiving chemotherapy and autophagic BC cell death [18]. In this study, we hypothesized that exosome-mediated delivery of miR-9-5p in the TAM resistant BC cells may mediate drug resistance and act via involvement of ADIPOQ.

## Materials and methods

### Ethics statement

All animal experiments were conducted in compliance with the guidelines of the Guangxi Medical University.

### Construction and culture of TAM-resistant MCF-7 (MCF-7/TAM) cell line

Initially, the MCF-7 cells (Cell Bank of the Chinese Academy of Sciences, Shanghai, China) were cultured in high-glucose Dulbecco’s modified Eagle’s medium (DMEM, Gibco, Grand Island, NY, USA) containing 10% fetal bovine serum (FBS, Gibco, Grand Island, NY, USA). MCF-7/TAM cells (Toronto Research Chemicals, North York, Ontario, Canada) were incubated in complete high-glucose DMEM containing 1.0 × 10^−7^ mol/L TAM for a period of 6 months. After establishment of drug resistance, the cells were cultured in high-glucose DMEM medium containing 10% FBS. The MCF-7/TAM cells were treated with 10 mg/mL or 20 mg/mL Gefitinib (AstraZeneca LP, Wilmington, DE, USA) for 48 h, followed by a regimen of incubation with 5% CO_2_ at 37 °C.

### Isolation and characterization of exosomes

Exosome free FBS was prepared by ultracentrifugation at 1 × 10^6^ g for 16 h (XL-100 K, Beckman Coulter, Fullerton, CA, USA) at 4 °C. After 48–72 h of incubation, the culture medium was harvested, and the exosomes were isolated by ultracentrifugation. Briefly, the cell culture medium was sequentially centrifuged at 300*g* for 10 min, at 2000*g* for 15 min and at 12,000*g* for 30 min to remove the floating cells and cell debris. Next, the medium was filtered using a 0.22 μm filter. The supernatant was then ultracentrifuged at 1 × 10^6^*g* for 2 h at 4 °C, and subjected to a second regimen of ultracentrifugation under similar conditions. Finally, the pellets were re-suspended using 100 mL of the phosphate buffered saline (PBS) and analyzed by NanoSight NS300 for concentration and size of exosomes.

### Cellular uptake of exosomes

A total of 200 pg exosomes were added to 1 mL of the Diluent C solution. Then, 4 μL of PKH67 fluorescent staining solution was added into another Eppendorf (EP) tube containing 1 mL of the Diluent C solution. These two solutions were mixed for 5 min, followed by the addition of 10 mL of 1% bovine serum albumin (BSA) to facilitate the binding of excessive staining solution. The mixed solution was centrifuged at 100,000*g* at 4 °C for 2 h, with removal of the supernatant. Finally, the solution was centrifuged at 100,000*g* at 4 °C for 2 h to isolate the exosome pellet, which was resuspended using complete medium. MCF-7 cells were incubated with PKH67-labeled exosomes and observed under a confocal microscope.

### Transmission electron microscopy (TEM)

The exosomes obtained by centrifugation of 400 mL of the medium at high-speed were fixed in 2% glutaraldehyde overnight at 4 °C. Then, exosomes were fixed with 1% OsO_4_ for 1 h, dehydrated in ethanol, and finally embedded in resin. The embedded sample was sliced using a microtome and saturated sodium periodate and 0.1 N hydrochloric acid were each added onto the sections. After 10 min, the sections were observed under a TEM (H-500, HITACHI, Tokyo, Japan).

### Cell transfection

Cells in the logarithmic growth phase were seeded in 6-well plates at a density of 6.0 × 10^5^ cells per well. According to the provided instructions of the Lipofectamine 2000 Transfection Kit, the MCF-7 cells and MCF-7/TAM cells were transfected with mimic and inhibitor, respectively. A 25 pmol mimic or inhibitor and 10 μL transfection reagent was added to each well to attain a final concentration of 10 pmol/mL, followed by incubation at 37 °C with 5% CO_2_. The cells were grouped as follows: the miR-9-5p mimic group (transfected with synthetic miR-9-5p mimic), miR-9-5p mimic-NC group (transfected with miR-9-5p mimic negative control sequence), miR-9-5p inhibitor group (transfected with miR-9-5p inhibitor), and miR-9-5p inhibitor-NC group (transfected with miR-9-5p inhibitor and negative control sequence). Each experiment was conducted 3 times independently. The cells were cultured for 48 h, and the exosomes were harvested for subsequent experimentation.

### Co-culture of exosomes with MCF-7 cells

Exosomes harvested from the transfected MCF-7 cells and MCF-7/TAM cells were co-cultured with MCF-7 cells for 48 h for subsequent experimentation. The MCF-7 cells were incubated with; exosomes extracted from the MCF-7 cells (MCF-7-exo group), exosomes extracted from the MCF-7 cells transfected with miR-9-5p mimic-NC ([MCF-7 + NC-mimic]-exo group), exosomes extracted from the MCF-7 cells transfected with miR-9-5p mimic ([MCF-7 + miR-9-5p mimic]-exo group), exosomes extracted from MCF-7/TAM cells (MCF-7/TAM-exo), exosomes extracted from the MCF-7/TAM cells transfected with miR-9-5p inhibitor-NC ([MCF-7/TAM + NC-inhibitor]-exo group), and exosomes extracted from the MCF-7/TAM cells transfected with miR-9-5p inhibitor ([MCF-7/TAM + miR-9-5p inhibitor]-exo group) respectively.

### Western blot analysis

The lysed samples were centrifuged at 7000*g* for 30 min at 4 °C to eliminate any cell debris. The supernatant was collected, and the total protein concentration was measured with a bicinchoninic acid kit. A total of 50 μg of the protein content was dissolved in 2× sodium dodecyl sulfate (SDS) loading buffer, and boiled at 100 °C. After 5 min, the samples were subjected to 10% SDS–polyacrylamide gel electrophoresis. The separated protein was transferred onto a polyvinylidene fluoride membrane, which was blocked using 5% skim milk powder at room temperature for 1 h. The membrane was incubated with the diluted primary antibodies to β-actin (ab8226, 1:1000), CD63 (ab216130, 1:5000), TSG101 (ab83, 1:5000), calnexin (ab22595, 1:5000) ADIPOQ (ab22554, 1:1000), cKIT (ab32363, 1:1000), CD44 (ab189524, 1:1000), and CD24 (ab179821, 1:1000). All antibodies were purchased from Abcam Inc. (Cambridge, UK). Next, the membrane was incubated with the horseradish peroxidase-labeled secondary antibody for 1 h. The protein bands were visualized using the enhanced chemiluminescence fluorescence detection kit (Cat. No. BB-3501, Amersham, Little Chalfont, Buckinghamshire, UK). The images were captured with a gel imager and photographed by a Bio-Rad Image Analysis System (Bio-Rad, Hercules, CA, USA), followed by quantitation with the Quantity One v4.6.2 software. The relative protein expression was expressed as the gray value of the corresponding protein band/the gray value of the β-actin protein band. The experiment was conducted 3 times independently.

### Cell counting kit-8 (CCK-8) assay

MCF-7 cells in the logarithmic growth phase were detached using 2.5 g/L trypsin. DMEM complete medium was added to prepare the single cell suspension, which was inoculated into the 96-well plates at 5 × 10^3^ cells/mL, followed by overnight incubation with CO_2_ at 37 °C. TAM at different concentrations was added for subsequent incubation of 48 h. A total of 10 μL freshly prepared CCK-8 solution (Dojdo, Kumamoto, Japan) was added to 100 μL of the incomplete medium and incubated for 4 h at 37 °C. The optical density (OD) value was measured at an excitation wavelength of 450 nm using a scanning spectrophotometer (Bio-Rad, Hercules, CA, USA). With the TAM concentration plotted on the x-axis and the OD_450_ value plotted on the y-axis, the growth curves of the cells were plotted. Besides, the difference in the half maximal inhibitory concentration (IC_50_) caused by TAM was calculated. All experiments were conducted three times independently.

### Annexin-V-fluorescein isothiocyanate (FITC)/propidium iodide (PI) double staining assay

MCF-7 cells in the logarithmic growth phase were seeded in six-well plates (approximately 5 × 10^4^ cells/well). After observing the adherence of cells to the wells, the cells were incubated with TAM of IC_50_ concentration for 24 h. The floating cells in the supernatant and the cells that adhered to the wells under normal growth were collected by centrifugation. The cell pellet was resuspended using 1× binding buffer and incubated with 5 μL Annexin-V-FITC and 10 μL PI for 15 min at room temperature in conditions devoid of light. The apoptosis rate was measured using a flow cytometer. Annexin-V-FITC positive cells represented early apoptosis, and the cells positive for both Annexin-V-FITC and PI represented advanced apoptosis. The experiment was conducted three times independently.

### PI staining for cell cycle

MCF-7 cells in the logarithmic growth phase were seeded in six-well plates at approximately 5 × 10^4^ cells/well. After observing the adherence of the cells to the well, the cells were incubated with TAM of IC_50_ concentration for 72 h. A single cell suspension of the MCF-7 cells was then prepared by detachment and was fixed overnight at − 20 °C in 1 mL of pre-cooled 75% alcohol. Then, PI and RNase were added for 30-min incubation at 37 °C in conditions devoid of light. A flow cytometer was used to detect the cell cycle distribution. The experiment was conducted three times independently.

### Quantification of gene expression

The Trizol method (Cat. No. 16096020, Thermo Fisher Scientific Inc., Waltham, MA, USA) was employed to extract the total RNA content. Then 5 μg of the total RNA content was reverse transcribed into cDNA using a quantitative real-time polymerase chain reaction (qRT-PCR) kit (ABI Company, Oyster Bay, N.Y., USA) according to the provided instructions. Reverse transcription and quantification of miRNA were performed using the miScript II RT kit (218161, QIAGEN, GmbH, Hilden, Germany) and the miScript SYBR Green PCR kit (218075, QIAGEN, GmbH, Hilden, Germany). The primer sequences of miR-9-5p, U6, ADIPOQ, and β-actin are presented in Table 1. U6 was adopted as the internal reference for miR-9-5p, while β-actin was adopted for other genes. The ratio of the expression of the target gene in the experimental group to that in the control group was estimated based on the 2^−ΔΔCt^ method. The experiment was conducted three times independently.

**Table 1:** Primer sequences for qRT-PCR

Gene	Forward (5′–3′)	Reverse (5′–3′)
miR-9-5p	GTGCAGGGTCCGAGGT	GCGCTCTTTGGTTATCTAGC
U6	AAAGCAAATCATCGGACGACC	GTACAACACATTGTTTCCTCGGA
ADIPOQ	TGTGTGTGTGGGGTCTGTCT	TGTGATGAAAGAGGCCAGAA
β-actin	GCACCACACCTTCTACAATG	TGCTTGCTGATCCACATCTG

### Microarray analysis

BC-related gene expression profiles were retrieved from the Gene Expression Omnibus (GEO) database (https://www.ncbi.nlm.nih.gov/geo/). The differential expression analysis was performed using the R language “limma” package, with |log foldchange (FC)| > 2 and *p* value < 0.05 as the outlining criteria to identify the differentially expressed genes (DEGs). A heat map of DEGs was constructed using the “pheatmap” package. The “clusterprofiler” package in the R language was employed for enrichment analysis of the metabolic pathways in the Kyoto Encyclopedia of Genes and Genomes (KEGG). A protein–protein interaction network was constructed using the String database (https://string-db.org). Regulatory miRNAs of ADIPOQ were predicted using a combination of the mirDIP database (http://ophid.utoronto.ca/mirDIP/index.jsp#r), the microRNA database (http://www.microrna.org/microrna/home.do?tdsourcetag=s_pcqq_aiomsg), the miRmap database (https://mirmap.ezlab.org), and the TargetScan database (http://www.targetscan.org/vert_71/).

### Luciferase activity assay

The 3′untranslated region (3′UTR) of the ADIPOQ gene was cloned with cDNA of the MCF7 cells as a template. Based on the binding site of miR-9-5p and ADIPOQ 3′UTR predicted by TargetScan, a site-directed mutagenesis was performed for the corresponding site. The 3′UTR wild-type (WT) was used as a template to construct an ADIPOQ 3′UTR reporter vector with mutation. The ADIPOQ-mutant (MUT) was constructed using the QuikChange Site-Directed Mutagenesis Kit (Stratagene, La Jolla, CA, USA). The mutation of miR-9-5p recognizing the ADIPOQ 3′UTR site sequence ACCAAAG was TGGTTTC. The primer sequences were recombined into the pNL1.1 vector (Promega, Madison, WI, USA) to transfect an amplified recombinant plasmid vector in *Escherichia coli* DH5α. The pRL-TK vector expressing Renilla luciferase was adopted as an internal reference to adjust the difference in cell number and transfection efficiency. The miR-9-5p mimic and miR-9-5p mimic-NC (4464084, ABI, Foster City, CA, USA) were co-transfected into the 293T cells with a dual luciferase reporter vector. The dual luciferase activity assay was performed in strict accordance with the instructions provided by Promega (Madison, WI, USA). Each experiment was conducted three times independently.

### Subcutaneous tumor formation in nude mice

In total, 40 female nude mice (4–5 weeks old) were injected subcutaneously with 1.5 mg/kg estradiol cetylpropionate 3 days prior to tumor implantation, after which they were injected once a week. All nude mice were housed in a specific pathogen free environment. Once cell confluence reached 70%, the MCF7 cells were detached, centrifuged, and suspended in PBS to isolate a cell suspension (1 × 10^8^ cells/mL). After anesthetizing the nude mice with 0.1% pentobarbital, the skin was incised along the third breast pad on the left side and dissected to the position of the breast pad. A total of 100 μL of the cell suspension was injected into the breast pad, after which the incision was sutured. After the tumor grew to 200 mm^3^, the nude mice were assigned into 5 groups: group 1: PBS; group 2: (MCF-7/TAM + miR-9-5p Inhibitor)-exo (exosomes extracted from MCF-7/TAM cells transfected with miR-9-p inhibitor); group 3: (MCF-7/TAM + miR-9-5p inhibitor-NC)-exo (exosomes extracted from MCF-7/TAM cells transfected with miR-9-5p inhibitor-NC); group 4: (MCF-7 + miR-9-5p mimic)-exo (exosomes of MCF-7 cells transfected with miR-9-5p mimic); group 5: (MCF-7 + miR-9-5p mimic-NC)-exo (exosomes of MCF-7 cells transfected with miR-9-5p mimic-NC). The transfected exosomes were injected for 7 times with an interval of 3 days into the tumor. TAM (20 mg/kg) or PBS was intragastrically administered into the nude mice twice a week, starting from the first injection of exosomes. Twenty-one days later, the nude mice were euthanized, and the tumor tissue was harvested. The tumors were measured and photographed. Subsequently, a proportion of the tumor was stored in liquid nitrogen, and the remaining tumor pieces and other organs were immersed in 4% paraformaldehyde overnight to prepare the wax blocks for immunohistochemistry. Tumor volume (V) was calculated with the measured length (L) and width (W) according to the following formula: V = (LW^2^)/2.

### Immunohistochemistry

Paraffin-embedded sections (4-μm in thickness) were dewaxed and subjected to ethylenediaminetetraacetic acid antigen repair. The sections were incubated with 3% H_2_O_2_ for 10 min at room temperature in order to terminate endogenous peroxidase activity and probed with antibody to ADIPOQ (ab22554, 1:1000, Abcam, Boston, MA, USA) at 4 °C overnight. The secondary antibody was added in a drop-wise manner and incubated at 37 °C for 20 min. After staining with 3,3′-diaminobenzidine (DAB), the sections were counterstained, dehydrated, permeabilized, and sealed.

### Terminal dUTP nick-end labeling (TUNEL) assay

The sections were dewaxed and hydrated. Five sections were incubated with 50 μL of 1% proteinase K in a 37 °C incubator for 30 min. Methanol containing 0.3% H_2_O_2_ was added to terminate endogenous peroxidase (POD) activity. TUNEL solution was added for incubation of the cells, in a humid chamber at 37 °C for 1 h in conditions devoid of light. Next, the cells were incubated with 50 μL Converter-POD at 37 °C for 30 min. Color development was conducted using 2% DAB with incubation for 15 min at room temperature. On appearance of brownish-yellow nuclei in the cells, the reaction was terminated using distilled water. After hematoxylin counterstaining, the sections were dehydrated, permeabilized using xylene, and sealed with neutral balsam. The cells were observed under an optical microscope.

### Three-dimensional (3-D) culture of MCF7 cells

MCF-7 cells were cultured as 3-D spheroids as described previously [19]. MCF-7 cells (3 × 10^4^) were incubated with the Matrigel (BD Biosciences, USA) that was added with DMEM/F12 containing 5% horse serum (GIBCO, USA), epidermal growth factor (EGF, 20 ng/mL), and 100 IU/mL penicillin/streptomycin under the condition of 5% CO_2_ and 37 °C. The Matrigel and DMEM/F12 was added every second day. The MCF7 cells were incubated with exosomes for 48 h and then were suspended at 1, 10, 100, 1000, and 10,000 cells/well. Sphere-forming ability was observed.

### Statistical analysis

All the data were processed using the SPSS 21.0 statistical software (IBM Corp, Armonk, NY, USA). The measurement data were expressed by mean ± standard deviation. The *t* test was adopted to compare paired data with normal distribution and equal variance between two groups. Unpaired data with normal distribution and equal variance were compared between two groups using the unpaired *t* test. Data comparison between multiple groups was performed using one-way analysis of variance (ANOVA) followed by the Tukey’s post hoc test. Data comparison between groups at different time points was performed by repeated measurement ANOVA with a Bonferroni’s post hoc test. In all statistical references, a value of *p *<0.05 was indicative of a statistically significant difference.

## Results

### Exosomes from MCF-7/TAM cells can be transferred into the parental MCF-7 cells

**Fig. 1:**
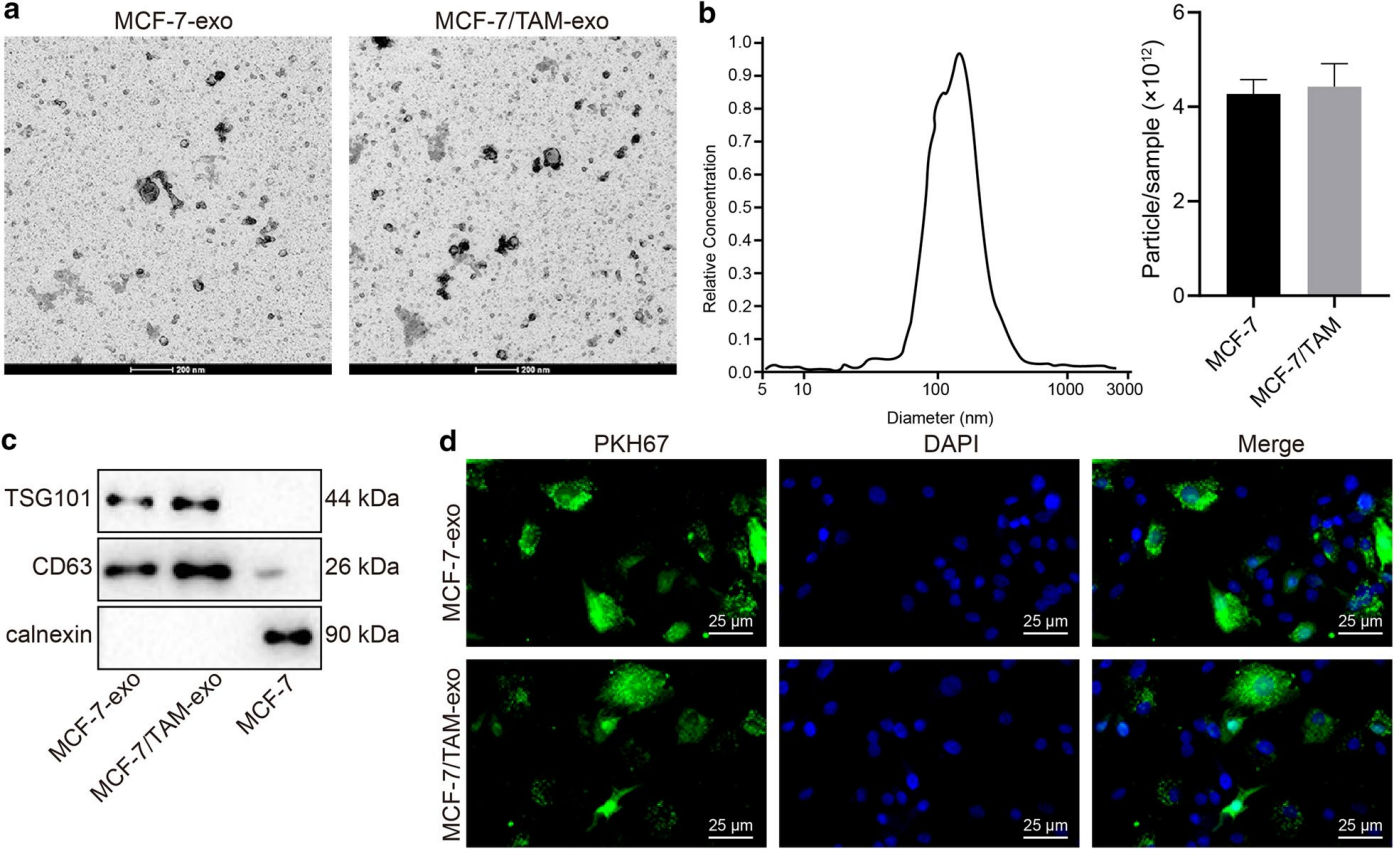
Exosomes from MCF-7/TAM cells can be transferred into the parental MCF-7 cells. **a** TEM observation of exosomes (scale bar: 200 nm). **b** Nanoparticle tracking analysis of exosome concentration and size. **c** The expression patterns of marker proteins (CD63 and TSG101) of exosomes in MCF-7-exo and MCF-7/TAM-exo detected by Western blot analysis (normalized to β-actin). **d** MCF-7-exo and MCF-7/TAM-exo (labeled with PKH67 dye) could be internationalized by parental MCF-7 cells (scale bar: 25 μm)

In order to examine the influence of exosomes on the drug resistance in BC cells, the exosomes were firstly isolated from the culture supernatant of MCF-7 cells and MCF-7/TAM cells and subsequently identified. TEM observation demonstrated the presence of round membrane-bound vesicles with a diameter of 30-100 nm (Fig. 1a). Observation of NanoSight NS300 indicated that isolated exosomes ranged from 60 to 120 nm and the concentration of exosomes in MCF-7 and MCF-7/TAM cells were 4.27 × 10^12^ particle/ml and 4.43 × 10^12^ particle/mL, respectively (Fig. 1b). Western blot analysis verified the expression of marker proteins CD63 and TSG101 in the exosomes (Fig. 1c). To investigate whether MCF-7/TAM-exo can be delivered to the sensitive cells and affect the resistant phenotype of sensitive cells, the MCF-7-exo and MCF-7/TAM-exo were co-cultured in the sensitive cells (MCF-7 cells) for 24 h. The results showed the capacity of sensitive cells to uptake exosomes from the Tamoxifen resistant cells (labeled by PKH67 staining) (Fig. 1d).

### Exosomes from MCF-7/TAM cells confers drug resistance in the parental MCF-7 cells

**Fig. 2:**
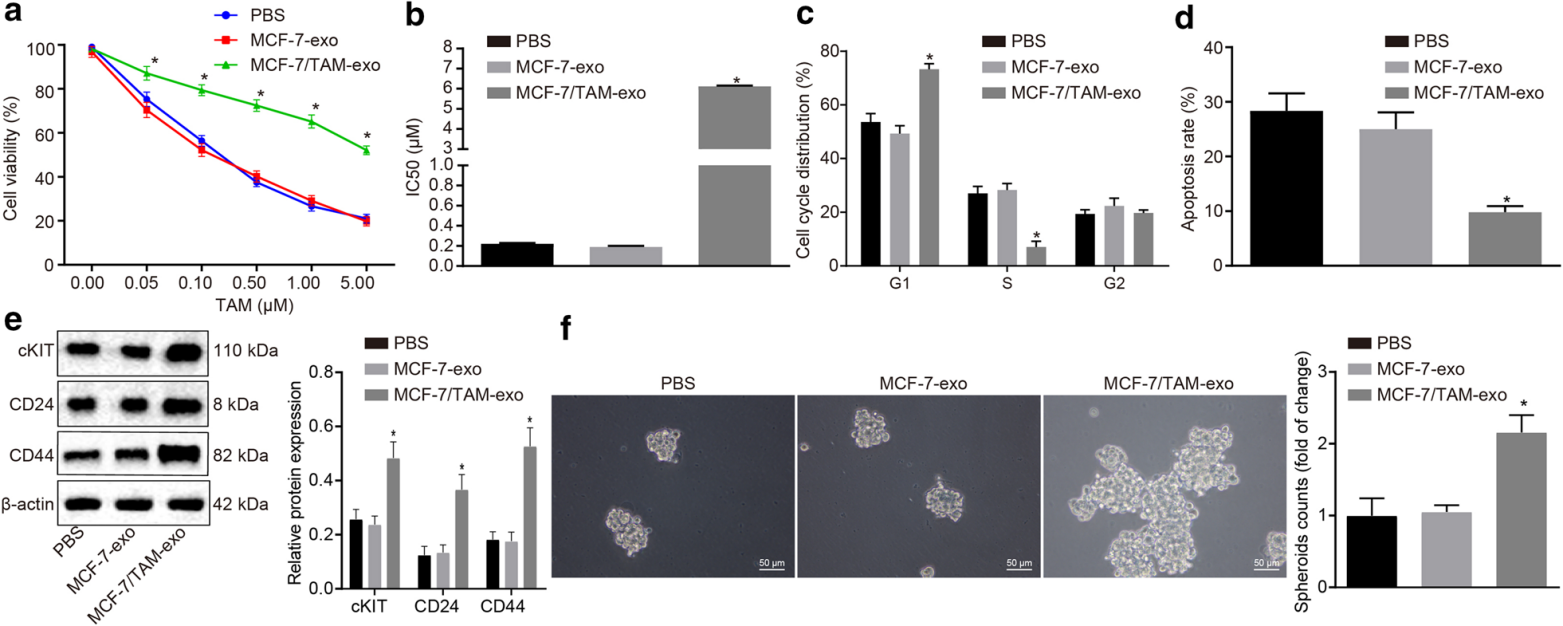
Exosomes from MCF-7/TAM cells confers drug resistance in the parental MCF-7 cells. **a** The cell viability of MCF-7 cells after TAM treatment determined by CCK-8. **b** IC_50_ values of MCF-7 cells treated with TAM. **c** The cell cycle distribution of MCF-7 in the presence of MCF-7/TAM-exo, analyzed by flow cytometry. **d** The apoptosis of MCF-7 cells in the presence of MCF-7/TAM-exo, analyzed by flow cytometry. **e** Western blot analysis of stemness markers KIT, CD44, and CD24 in MCF-7 cells in the presence of MCF-7/TAM-exo. **f** Sphere-forming abilities of MCF-7 cells in the presence of MCF-7/TAM-exo were examined by 3-D cell culture (scale bar: 50 μm). *p <0.05. All experiments were conducted three times independently

CCK-8 assay (Fig. 2a, b) was conducted to evaluate whether MCF-7/TAM-exo increased the resistance of MCF-7 cells to TAM. MCF-7 cells were treated with PBS, MCF-7-exo or MCF-7/TAM-exo for 9 days, each, after which TAM of variable concentrations was added. After 72 h of culture, the OD value was measured using a microplate reader, after which the cell viability curve was plotted. In comparison to treatment with PBS and MCF-7-exo, the cell viability of MCF-7 cells co-cultured with MCF-7/TAM-exo was significantly enhanced and IC_50_ was significantly elevated under TAM treatment (*p *<0.05). Besides, flow cytometry was conducted to detect the cell cycle distribution (Fig. 2c) of MCF7 cells co-cultured with exosomes and the apoptosis rate (Fig. 2d) after 10 μM of TAM treatment, so as to assess that the drug resistance conferred. Relative to the treatment of PBS and MCF-7-exo, the MCF-7 cells co-cultured with MCF-7/TAM-exo demonstrated an increased number of cells in the G1 phase and a decreased number of cells in the S phase (*p *<0.05), accompanied by a reduced apoptotic rate (*p *<0.05). We examined the effects of MCF-7/TAM-exo on the stemness of MCF-7 cells. It revealed that MCF-7/TAM-exo promoted the stemness of MCF-7 cells as evidenced by increased expressions of stemness markers cKIT, CD44, and CD24 when comparable to PBS and MCF-7-exo (Fig. 2e). Accordingly, results of 3-D cell culture displayed that MCF-7/TAM-exo enhanced sphere-forming abilities of MCF-7 cells when comparable to PBS and MCF-7-exo (Fig. 2f). These results indicate that MCF-7/TAM-exo could increase the resistance of MCF-7 cells to TAM.

### Microarray-based analysis identifies ADIPOQ and miR-9-5p as the study subjects

**Fig. 3:**
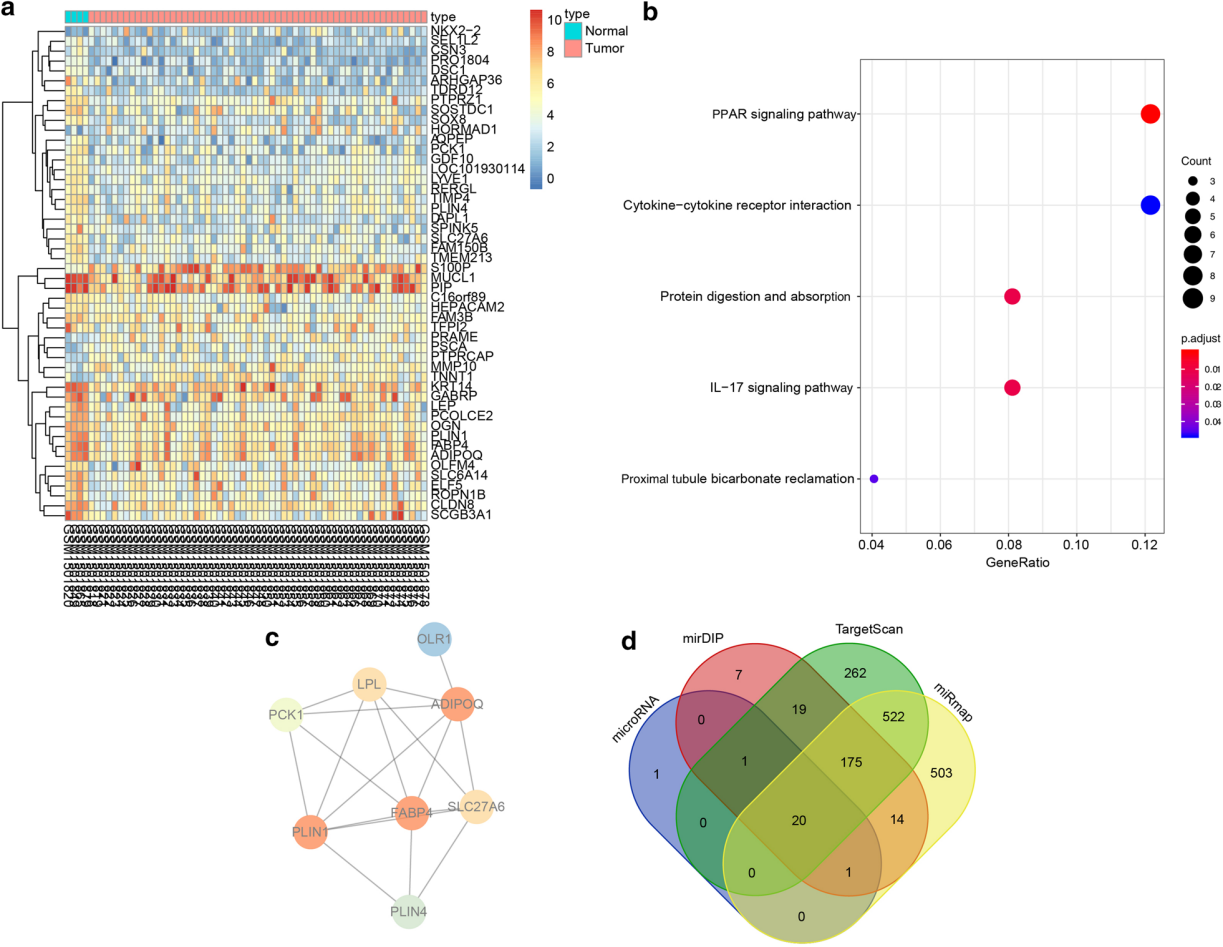
ADIPOQ and miR-9-5p is involved in the drug resistance of BC. **a** Heat map of BC-related DEGs in different expression profiles. The x-axis indicates the sample number, the y-axis indicates the gene name, and the left dendrogram indicates the gene expression cluster. Each small square in the panel indicates the expression of a gene in one sample. **b** Enrichment analysis of DEGs in KEGG metabolic pathways. The x-axis refers to GeneRatio, the y-axis refers to KEGG signaling pathways, and the right histogram represents color gradation. **c** Protein–protein interaction network of the DEG in the PPAR signaling pathway. Each circle represents a gene and the circle color represents the core degree of the DEG in the interaction network. Darker color means higher core degree. **d** Prediction of upstream regulatory miRNAs of ADIPOQ. Four ellipses refer to the results predicted from the four databases and the central section refers to the intersecting of the prediction results

In order to identify the genes involved in the effect of exosomes on the drug resistance of BC cells, we explored the GEO database to retrieve the BC-related expression profile GSE61304, which consisted of 4 normal samples and 58 BC samples. Differential analysis of gene expression in the BC samples and normal control samples in this expression profile was performed, which identified 176 DEGs (Fig. 3a). Functional enrichment analysis revealed that the DEGs were principally enriched in several KEGG signaling pathways like the PPAR signaling pathway, which elicited the highest enrichment level (Fig. 3b). Besides, the corresponding DEGs in the PPAR signaling pathway were subjected to protein–protein interaction analysis, which revealed that ADIPOQ, FABP4, and PLIN1 were the core genes (Fig. 3c). Therefore, the significant functions of these three genes were retrieved, which showed that ADIPOQ could negatively regulate tumor growth [18] and was associated with the therapeutic effects of TAM [20]. In order to further investigate the upstream regulatory mechanism of ADIPOQ, the regulatory miRNAs of ADIPOQ were predicted using multiple bioinformatic databases such as TargetScan and the intersecting results were determined (Fig. 3d) to classify 20 miRNAs as common. Among them, miR-9-5p was evidently carried by exosomes and thus exerted effects [21, 22]. These results and findings suggest that miR-9-5p, via the delivery of exosomes, might target and regulate ADIPOQ to influence the effects of TAM on BC.

### ADIPOQ is a target gene of miR-9-5p

**Fig. 4:**
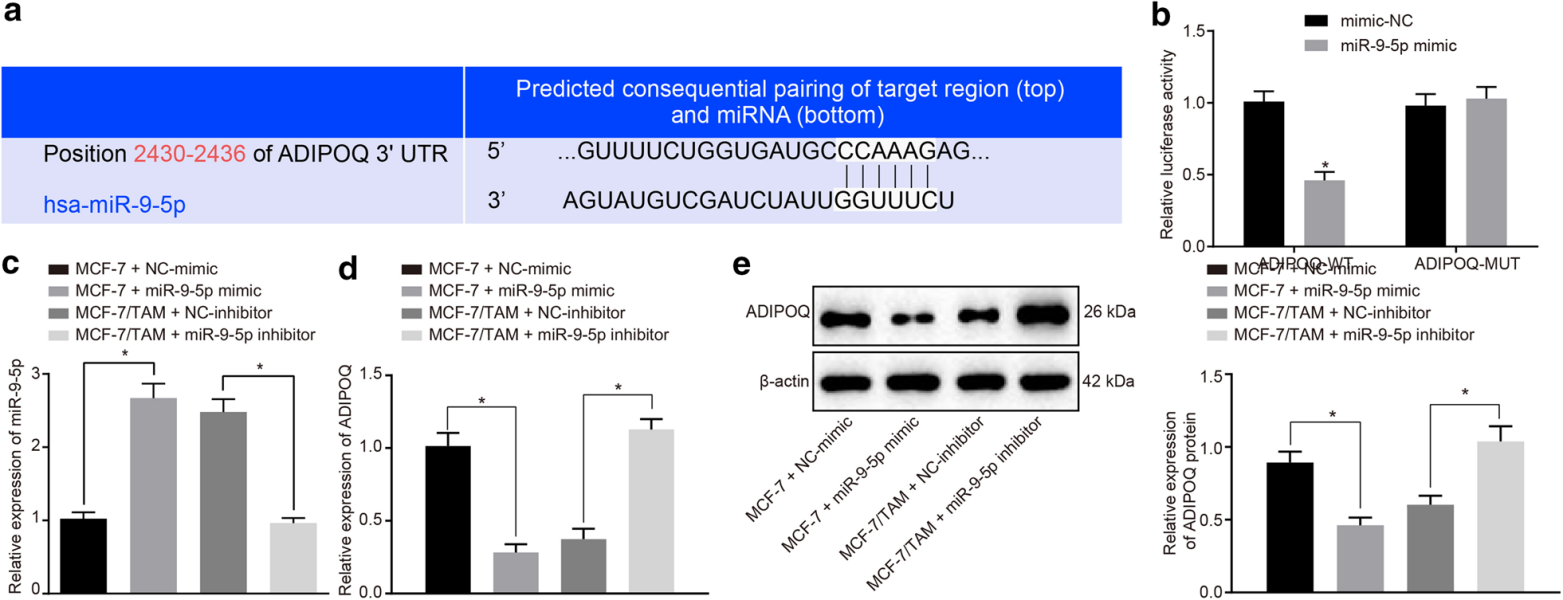
miR-9-5p targets and negatively regulates ADIPOQ. **a** The prediction of binding sites between miR-9-5p and ADIPOQ. **b** Quantitation of the luciferase activity assay. **c** The expression pattern of miR-9-5p in each group measured by RT-qPCR. **d** The mRNA expression pattern of ADIPOQ in the cells of each group measured by RT-qPCR. **e** The protein expression pattern of ADIPOQ in cells of each group evaluated by Western blot analysis. *p <0.05. Each experiment was conducted three times independently

The binding site between miR-9-5p and ADIPOQ was predicted using the biological prediction website TargetScan (http://www.targetscan.org) (Fig. 4a). The dual luciferase reporter assay was adopted to ascertain ADIPOQ as a target of miR-9-5p. In response to the site-directed mutagenesis on the binding sites of miR-9-5p and ADIPOQ-WT, the luciferase activity of 293T cells in the miR-9-5p mimic and ADIPOQ-WT co-transfection group had decreased by 50% (*p *<0.05), while no significant changes were observed in the luciferase activity in the miR-9-5p mimic and ADIPOQ-MUT transfection group (Fig. 4b). This result indicates that miR-9-5p can directly regulate the ADIPOQ gene. Simultaneously, we further verified the results by transfecting miR-9-5p mimic and inhibitor into the MCF-7 or MCF-7/TAM cells. After transient transfection of miR-9-5p mimic in the MCF-7 cells, the miR-9-5p expression pattern was significantly increased (Fig. 4c, *p *<0.05), while the mRNA and protein expression patterns of ADIPOQ were significantly reduced (Fig. 4d, e, *p *<0.05), as compared to mimic-NC. After transient transfection of miR-9-5p inhibitor in the MCF-7/TAM cells, the expression pattern of miR-9-5p was significantly decreased (Fig. 4c, *p *<0.05), while the mRNA and protein expression patterns of ADIPOQ were considerably increased (Fig. 4d, e, *p *<0.05), as compared to inhibitor-NC. The results indicated that the expression pattern of miR-9-5p could be effectively regulated in the cells by transient transfection of miR-9-5p mimic or miR-9-5p inhibitor, and miR-9-5p targeted and regulated the expression pattern of ADIPOQ.

### MiR-9-5p promotes TAM resistance in the parental MCF-7 cells

**Fig. 5:**
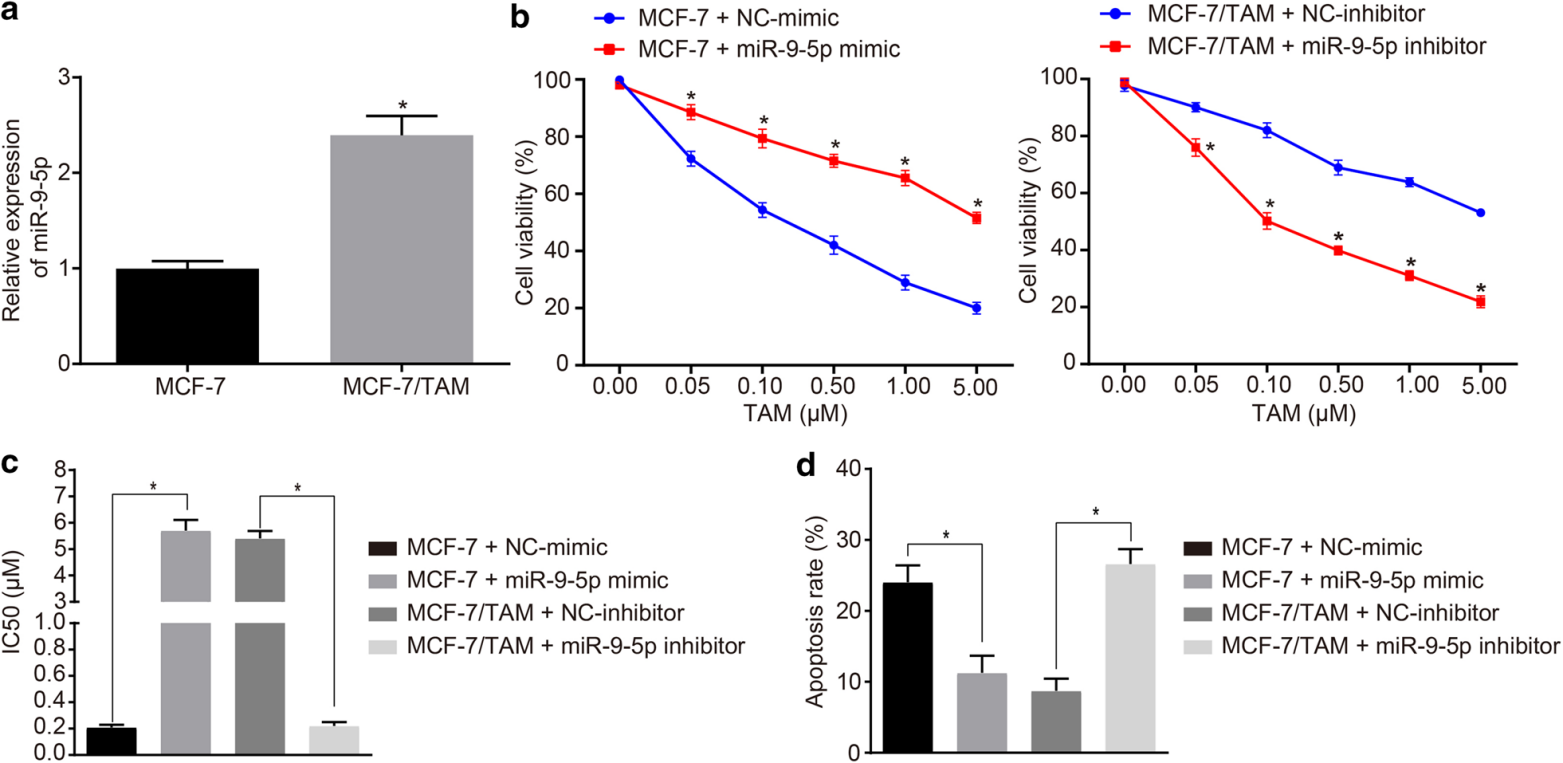
MiR-9-5p promotes TAM resistance in the parental MCF-7 cells. **a** The miR-9-5p expression in MCF-7 and MCF-7/TAM cell lines measured by RT-qPCR. **b** The cell viability of cells in response to miR-9-5p mimic or miR-9-5p inhibitor determined by CCK-8. **c** Quantitation of IC_50_ value of cells in response to miR-9-5p mimic or miR-9-5p inhibitor. **d** Flow cytometric detection of apoptosis rate of cells transfected with miR-9-5p mimic or miR-9-5p inhibitor. *p <0.05. Each experiment was conducted three times independently

To further investigate the role of miR-9-5p in BC cell resistance, the expression pattern of miR-9-5p in the MCF-7 and MCF-7/TAM cells was examined. The expression pattern of miR-9-5p was significantly increased in the MCF-7/TAM cells (Fig. 5a, *p *<0.05). We further conducted experiments to determine sensitivity of the transfected cells, with diluted TAM at different concentrations for treatment of the transfected cells for 48 h. The OD value of the transfected cells was detected using a CCK-8 kit, and the IC_50_ value of cells was calculated using the SPSS software. In comparison with mimic-NC, the transient transfection of miR-9-5p mimic in MCF-7 cells decreased the cell inhibition rate, accompanied by increased IC_50_ (*p *<0.05), along with a decreased inhibition rate in a dose-dependent manner (Fig. 5b, c). In consistency, the transient transfection of miR-9-5p inhibitor in MCF-7/TAM cells exercised conflicting results (*p *<0.05; Fig. 5b, c). To investigate the effect of miR-9-5p on the apoptosis of MCF-7 cell line, flow cytometry was adopted. After transient transfection of miR-9-5p mimic into the MCF-7 cells, the apoptosis rate of cells was significantly reduced as compared to mimic-NC (Fig. 5d, *p *<0.05). In the presence of transient transfection of miR-9-5p inhibitor, the cell apoptosis rate was significantly elevated relative to the inhibitor-NC (Fig. 5d, *p *<0.05). The aforementioned results indicated that miR-9-5p could inhibit MCF-7 cells apoptosis and promote drug resistance.

### Exosomes carrying miR-9-5p down-regulates the expression of ADIPOQ in BC cells

To validate whether exosomal miR-9-5p mediated the resistance by the regulation of ADIPOQ expression, we delivered the miR-9-5p mimic and inhibitor into the isolated exosomes from the MCF-7 and MCF-7/TAM cells followed by co-culture with the MCF-7 cells, in order to verify whether exosomes affect BC cells by carrying miR-9-5p.

**Fig. 6:**
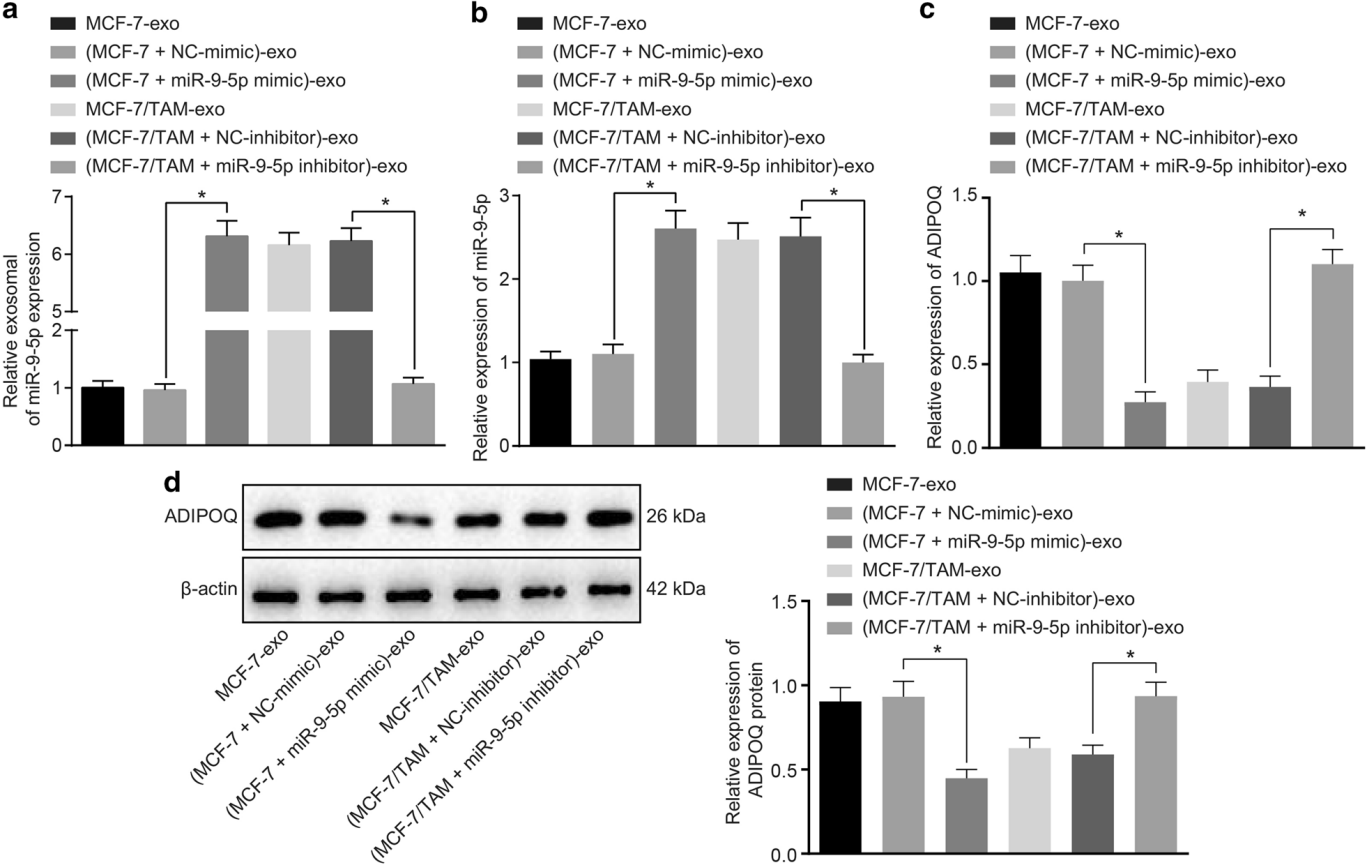
Exosomes carrying miR-9-5p down-regulate the expression pattern of ADIPOQ in BC cells. **a** miR-9-5p expression in (MCF-7 + miR-9-5p mimic)-exo and (MCF-7/TAM + miR-9-5p inhibitor)-exo measured by RT-qPCR. **b** q expression pattern of miR-9-5p in the MCF-7 recipient cells after co-culture of (MCF-7 + miR-9-5p mimic)-exo or (MCF-7/TAM + miR-9-5p inhibitor)-exo measured by RT-qPCR. **c** The mRNA expression pattern of ADIPOQ in the MCF-7 recipient cells after co-culture of (MCF-7 + miR-9-5p mimic)-exo or (MCF-7/TAM + miR-9-5p inhibitor)-exo measured by RT-qPCR. **d** The protein expression pattern of ADIPOQ in the MCF-7 cells after co-culture of (MCF-7 + miR-9-5p mimic)-exo or (MCF-7/TAM + miR-9-5p inhibitor)-exo evaluated by Western blot analysis. *p <0.05. Each experiment was conducted three times independently

As shown in Fig. 6a, the expression pattern of miR-9-5p in (MCF-7 + miR-9-5p mimic)-exo was significantly higher compared to that of (MCF-7 + NC-mimic)-exo (*p *<0.05). The miR-9-5p expression pattern of (MCF-7/TAM + miR-9-5p inhibitor)-exo was lower than that of (MCF-7/TAM + NC-inhibitor)-exo (*p *<0.05). Conjointly, the results suggested that exosomes could transfer miR-9-5p. After co-culture of these exosomes with MCF-7 cells, the expression pattern of miR-9-5p and ADIPOQ in the recipient MCF-7 cells were measured. Increased miR-9-5p expression and reduced mRNA and protein expression pattern of ADIPOQ were identified in the MCF-7 cells co-cultured with (MCF-7 + miR-9-5p mimic)-exo, compared with the MCF-7 cells co-cultured with (MCF-7 + NC-mimic)-exo (Fig. 6b–d, *p *<0.05). Moreover, after co-culture of MCF-7 cells with (MCF-7/TAM + miR-9-5p inhibitor)-exo, the expression pattern of miR-9-5p had reduced by 70%, whereas the mRNA and protein expression patterns of ADIPOQ were elevated as compared to co-culture with (MCF-7/TAM + NC-inhibitor)-exo (Fig. 6b–d, *p *<0.05). These results indicated that the transfection of miR-9-5p mimic and inhibitor into the MCF-7 and MCF-7/TAM-derived exosomes could effectively regulate the expression pattern of miR-9-5p in the recipient MCF-7 cells, accompanied by the regulation of ADIPOQ.

### MiR-9-5p dictates drug resistance conferred by exosomes isolated from MCF-7/TAM cells in the parental MCF-7 cells

**Fig. 7:**
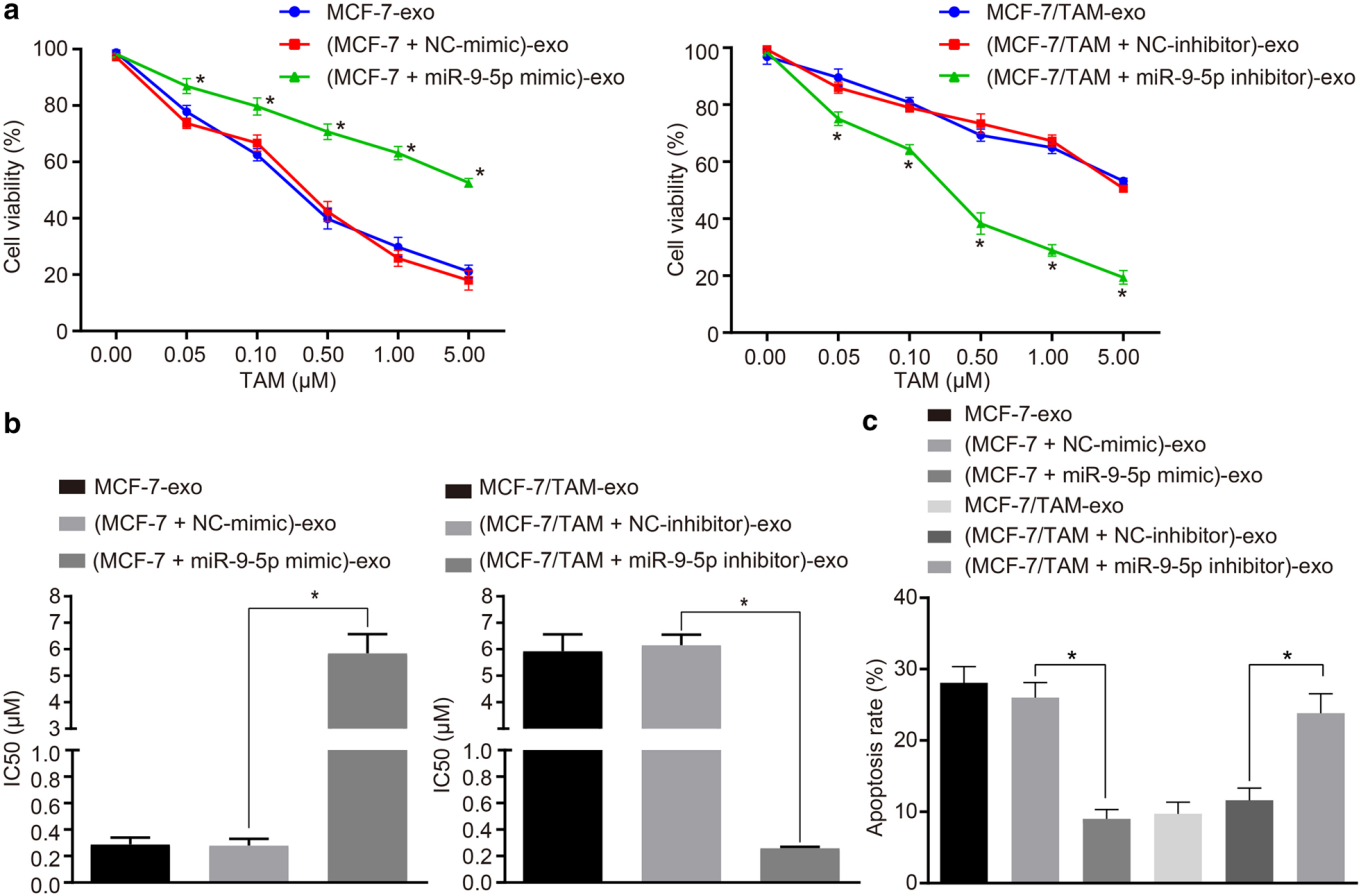
MiR-9-5p dictates drug resistance conferred by exosomes isolated from MCF-7/TAM cells in the parental MCF-7 cells. **a** The viability of MCF-7 recipient cells after co-culture with (MCF-7 + miR-9-5p mimic)-exo or (MCF-7/TAM + miR-9-5p inhibitor)-exo determined by CCK-8. **b** The quantitation of IC_50_ of MCF-7 recipient cells after co-culture. **c** Flow cytometric detection of the apoptosis of MCF-7 recipient cells after co-culture with (MCF-7 + miR-9-5p mimic)-exo or (MCF-7/TAM + miR-9-5p inhibitor)-exo. *p <0.05. Each experiment was conducted three times independently

To further investigate the role of (MCF-7 + miR-9-5p mimic)-exo and (MCF-7/TAM + miR-9-5p inhibitor)-exo in respect to drug resistance of BC cells, the sensitivity of MCF-7 cells with different transfections was strenuously evaluated. The MCF-7 cells elicited a reduced cell inhibition rate and increased IC_50_ after co-culture of (MCF-7 + miR-9-5p mimic)-exo, relative to the co-culture of (MCF-7 + NC-mimic)-exo (Fig. 7a, b, *p *<0.05), and the inhibition rate was abrogated in a dose-dependent manner (Fig. 7a). These results indicated that the up-regulation of exosomal miR-9-5p expression could improve the resistance of MCF-7 cells to TAM. Furthermore, the same procedure was adopted to examine the effect of miR-9-5p inhibitor on the sensitivity of MCF-7 cells to TAM. The MCF-7 cells co-cultured with (MCF-7/TAM + miR-9-5p inhibitor)-exo led to increased cell inhibition rate and reduced IC_50_ relative to the co-culture of (MCF-7/TAM + NC-inhibitor)-exo (Fig. 7a, b, *p *<0.05), with the inhibition rate eliciting a dose-dependent relationship (Fig. 7a). These findings suggested that exosomes alter the sensitivity of MCF-7 cells to TAM by exosomal miR-9-5p. Meanwhile, flow cytometry was adopted to detect the apoptosis of the transfected cells. The MCF-7 cells co-cultured with (MCF-7 + miR-9-5p mimic)-exo exhibited a reduced apoptosis rate, as compared with (MCF-7 + NC-mimic)-exo (Fig. 7c, *p *<0.05). After co-culture of MCF-7 cells with (MCF-7/TAM + miR-9-5p inhibitor)-exo, the apoptosis rate was increased as compared to (MCF-7/TAM + NC-inhibitor)-exo (Fig. 7c, *p *<0.05). These results indicated the ability of exosomes to transfer miR-9-5p into the MCF-7 cells, and subsequent initiate the inhibition of apoptosis and promotion of drug resistance.

### MiR-9-5p dictates drug resistance conferred by exosomes isolated from MCF-7/TAM cells by regulating ADIPOQ in vivo

**Fig. 8:**
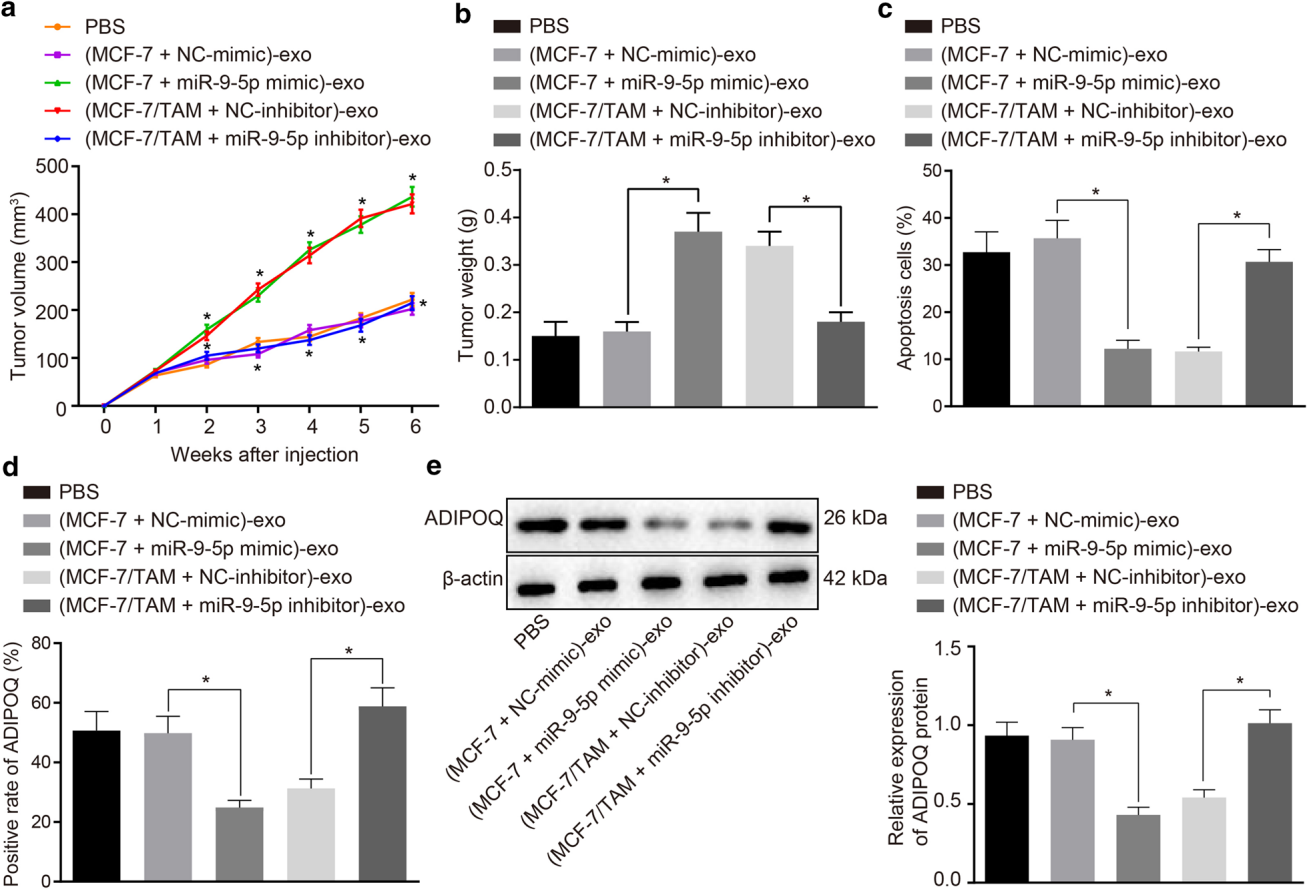
MiR-9-5p dictates drug resistance conferred by exosomes isolated from MCF-7/TAM cells by regulating ADIPOQ in vivo. **a** The growth curve of tumors with multipoint injection of exosomes. **b** The quantitation of tumor weight changes after multipoint injection of exosomes into the tumor. **c** The apoptosis of tissues in different groups detected by TUNEL staining. **d** Protein expression of ADIPOQ in tumors of different group. **e** Protein expression of ADIPOQ determined by Western blot analysis. *p <0.05. n = 8

To further investigate the effect of exosomal miR-9-5p on tumor resistance to TAM in vivo, the MCF-7 orthotopic tumors implanted in the nude mice were ascertained. When the tumor grew to 100 mm^3^, the exosomes were injected into the tumors at multiple sites, once every 3 days, for a total of 7 trials. TAM was weekly administered intragastrically to simulate the standard administration process. The results showed that the tumor volume and weight of the nude mice injected with (MCF-7 + miR-9-5p mimic)-exo were significantly higher compared to those injected with (MCF-7 + NC-mimic)-exo. Besides, the tumor volume and weight of the nude mice injected with (MCF-7/TAM + miR-9-5p inhibitor)-exo were significantly lower than those with (MCF-7/TAM + NC-inhibitor)-exo (Fig. 8a–c). TUNEL staining (Fig. 8d) was employed so as to assess MCF-7 cell apoptosis. The results showed that the cell apoptosis in tumors of nude mice injected with (MCF-7 + miR-9-5p mimic)-exo was significantly reduced relative to those injected with (MCF-7 + NC-mimic)-exo. Besides, the cell apoptosis in tumors of nude mice injected with (MCF-7/TAM + miR-9-5p inhibitor)-exo was significantly higher than those injected with (MCF-7/TAM + NC-inhibitor)-exo. In addition, immunohistochemistry and Western blot analysis were employed to measure the protein expression patterns of ADIPOQ. ADIPOQ was predominantly expressed in the cytoplasm. The protein expression pattern of ADIPOQ in the tumors of nude mice injected with (MCF-7 + miR-9-5p mimic)-exo was significantly lower than those injected with (MCF-7 + NC-mimic)-exo. Besides, the expression pattern of the ADIPOQ protein in the tumors of nude mice injected with (MCF-7/TAM + miR-9-5p inhibitor)-exo was significantly higher compared to those injected with (MCF-7/TAM + NC-inhibitor)-exo (Fig. 8e, f). In summary, exosomal miR-9-5p could evident inhibit the expression pattern of ADIPOQ in vivo and mediate tumor cell resistance to TAM.

## Discussion

BC is one of the most frequently diagnosed fatal cancers and persists as the leading cause of cancer associated mortality amongst women [23]. TAM is commonly adopted for the treatment of BC, however the drug resistance has been significant to unsatisfactory treatment outcomes [8, 24]. Research has well established the ability of exosomal miRNAs to shuttle through cells in order to transfer genetic material, and thus, play pivotal roles in tumorigenesis and drug resistance [25]. Moreover, an existing study elicited altered tumor growth consequent of intravenously injected exosomal miRNA to the xenograft BC tissues [26]. Our chief findings indicated that exosomal miR-9-5p could confer drug resistance in BC cells to TAM by regulating ADIPOQ.

The exosomes secreted by the TAM-resistant BC cells (MCF-7/TAM cells) could evidently augment the resistance of MCF-7 cells to TAM, as imitated by repressed cell apoptosis and cell cycle arrest. These findings are broadly aligned with existing evidence eliciting that drug-resistant BC cells could confer the resistance capacity to drug-sensitive cells by secreting exosomes, which partly function by shuttling of specific miRNAs [27]. In consistency with this, Santos et al. demonstrated that exosomes-mediated transfer of miRNAs could affect drug resistance in BC cells, thereby surfacing as candidate biomarkers for the investigation of BC progression and therapy [28]. In the current study, our findings revealed that miR-9-5p could repress MCF-7 cell apoptosis and promote the resistance to TAM. The expression of miR-9 has been implicated with clinicopathological significance in BC metastasis, whereby its up-regulation can facilitate and hasten the progression of invasive tumors [15]. In addition, an existing study demonstrated an association between miR-9-5p expression in BC and the status of hormone receptors, thus influencing the survival conditions of patients [29]. Besides, an increased expression of miR-9-5p was indicative of a poor prognosis in BC patients with the involvement of oestrogen-regulated pathways [30]. Another study suggested that the miR-9-5p expression in the nephrectomy samples could serve as a potential indicator for predicting resistance to first-line therapy in patients with metastatic renal cell carcinoma [31].

Notably, the MCF-7 cells co-cultured with MCF-7/TAM cell-derived exosomes transfected with miR-9-5p mimic demonstrated radically facilitated cell viability and reduced apoptosis, accompanied by improved resistance to TAM. Cancer cell-derived exosomes containing miRNAs can extensively induce the metastatic potential in recipient cells, during the process of invasion [32]. Therefore, a comprehensive understanding of the role of miRNA in regulation of the tumor microenvironment via exosomes may help develop novel therapeutic agents. For example, exosome-mediated transfer of miR-10b could promote cell invasion in BC by inhibiting the target genes of HOXD10 and KLF4 [33]. Additionally, an increasing number of studies indicating that exosomal miRNAs may either facilitate or hinder tumor progression by improving drug resistance, and metastatic potential, with regulation of the delivery of tumor-promoting or tumor-suppressive exosomal miRNAs which are regarded as effective treatment strategies [34, 35].

Importantly, a combination of bioinformatic analysis and luciferase activity assay verified that ADIPOQ was targeted and negatively regulated by miR-9-5p. ADIPOQ has been proposed to serve as a putative target gene of miR-3634 and was correlated to cell invasion in BC [36]. Supporting findings from an existing study exhibited a correlation between miR-9 expression and the malignant phenotype and chemoresistance of bladder cancer by targeting LASS2 [37]. An overarching finding of this study was observing that exosomal miR-9-5p lowered the ADIPOQ expression to improve the drug resistance in vivo and in vitro. Earlier reports have documented the aggravating role of miR-9 in malignancy. The up-regulation of miR-9 could facilitate metastasis formation in highly malignant cells [38]. MiR-9-5p elicited potential as a catalyst for invasiveness and metastasis by targeting TGFBR2 in non-small cell lung cancer [16]. In the light of existing evidence, our experiments have delineated the exosomal miR-9-5p-ADIPOQ interaction as mechanism to alter drug resistance in BC.

**Fig. 9:**
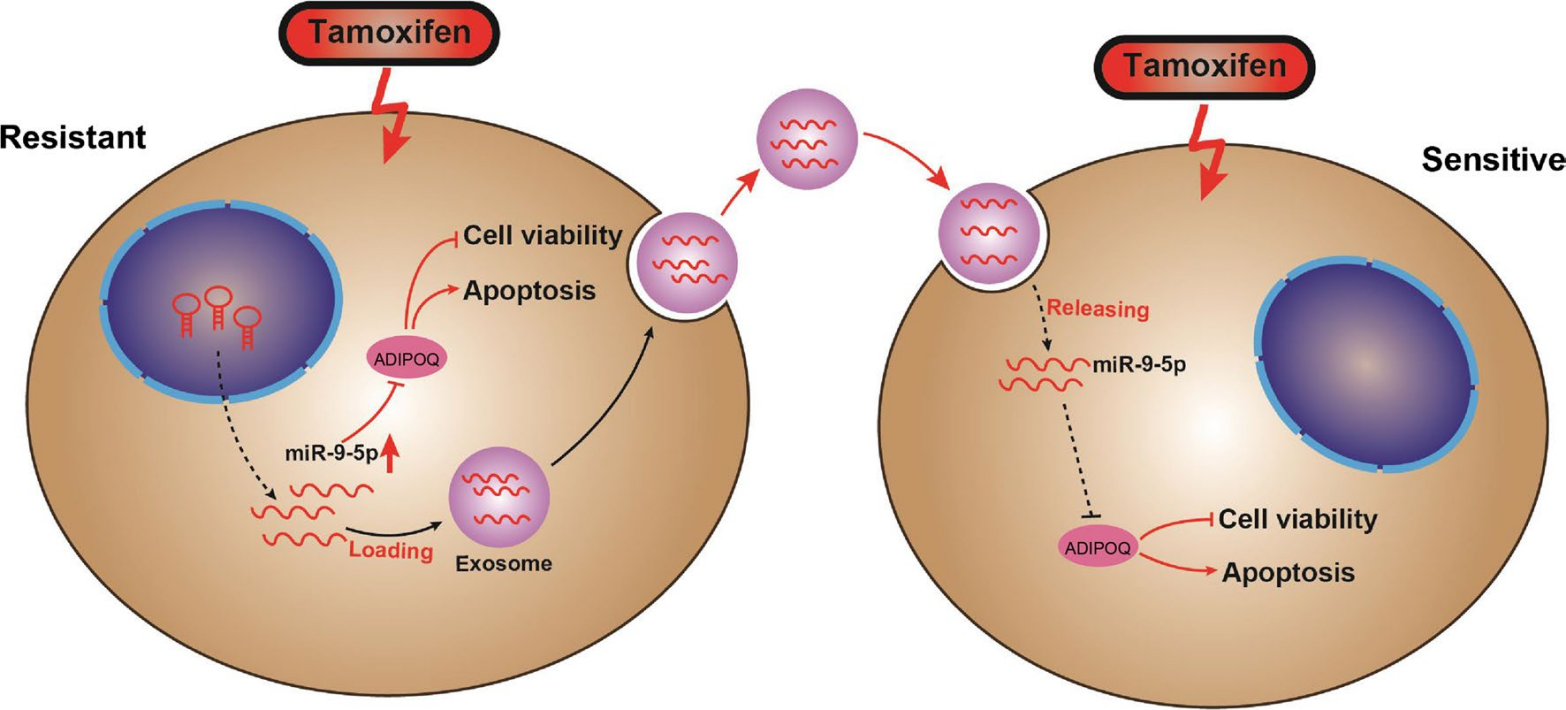
The schematic representation of the molecular mechanism miR-9-5p in TAM-resistant BC cells. In TAM-resistant BC cells, miR-9-5p promotes TAM resistance by negatively regulating ADIPOQ, whereby it promotes BC cell viability and inhibits apoptosis. In addition, miR-9-5p can be packaged into the exosomes derived from TAM-resistant BC cells, and transferred into the TAM-sensitive BC cells

Based on the aforementioned evidence, we conclude that exosomal transfer of miR-9-5p facilitated the resistance of BC cells to TAM by negatively regulating ADIPOQ, and this interaction network could provide an insight on a promising target for the prevention and management of drug resistance in BC (Fig. 9). However, due to the preclinical stage of the current research, further investigations are warranted to elucidate the supporting mechanisms for subsequent clinical translation.

## Data Availability

The datasets used or analysed during the current study are available from the corresponding author on reasonable request.
