# Correction to: Exosomes from tamoxifen-resistant breast cancer cells transmit drug resistance partly by delivering miR-9-5p

**DOI:** 10.1186/s12935-021-01944-6

**Published:** 2021-04-29

**Authors:** Jianhui Liu, Shaoliang Zhu, Wei Tang, Qinghua Huang, Yan Mei, Huawei Yang

**Affiliations:** 1 The First Department of Breast Surgery, Guangxi Medical University Cancer, Hospital, Nanning, 530021 People’s Republic of China; 2 Department of Hepatobiliary Surgery, Guangxi Medical University Cancer Hospital, No.71, Hedi Road, Nanning, 530021 Guangxi People’s Republic of China

## Correction to: Cancer Cell Int (2021) 21:55 https://doi.org/10.1186/s12935-020-01659-0

Following the publication of the original article [1], we were notified of an error in Fig. 4.

The corrected Fig. 4 can be found below.

**Fig. 4:**
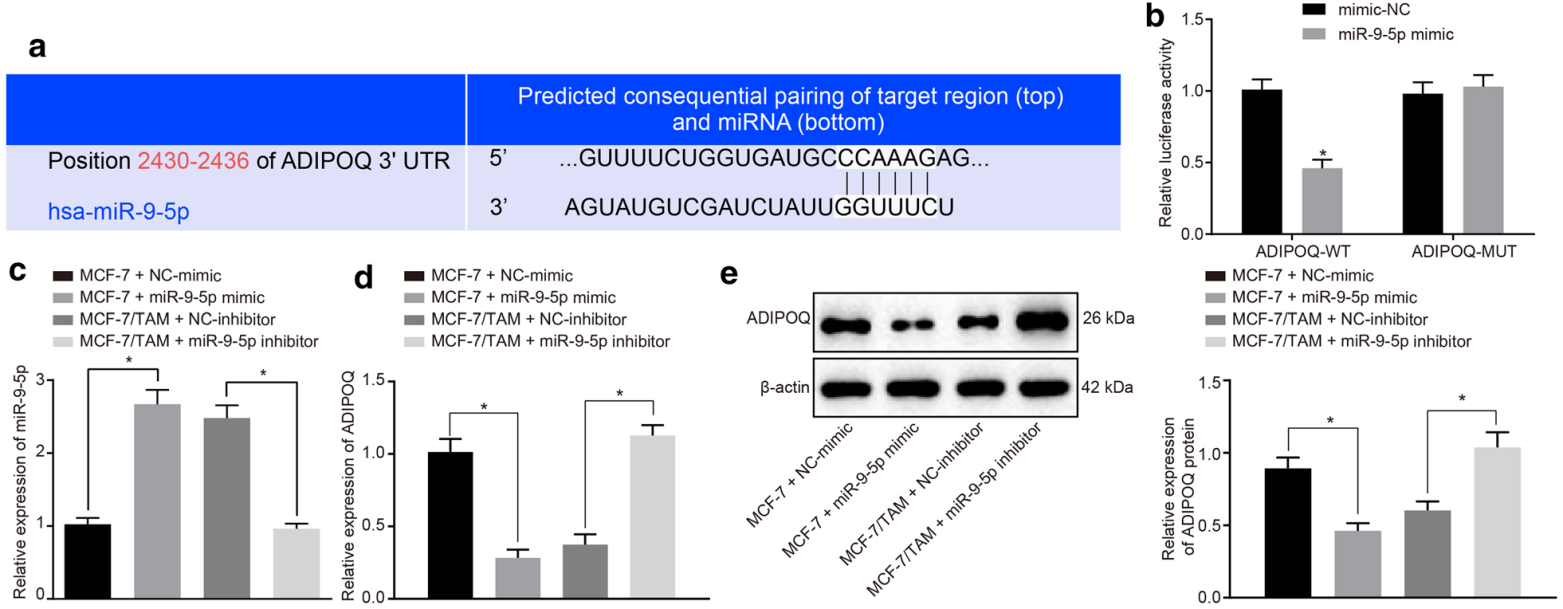
miR-9-5p targets and negatively regulates ADIPOQ. **a** The prediction of binding sites between miR-9-5p and ADIPOQ. **b** Quantitation of the luciferase activity assay. **c** The expression pattern of miR-9-5p in each group measured by RT-qPCR. **d** The mRNA expression pattern of ADIPOQ in the cells of each group measured by RT-qPCR. **e** The protein expression pattern of ADIPOQ in cells of each group evaluated by Western blot analysis. *p < 0.05. Each experiment was conducted three times independently

The original article has been corrected.
