# Correction to: Exosomes from tamoxifen-resistant breast cancer cells transmit drug resistance partly by delivering miR-9-5p

**DOI:** 10.1186/s12935-021-02370-4

**Published:** 2021-12-14

**Authors:** Jianhui Liu, Shaoliang Zhu, Wei Tang, Qinghua Huang, Yan Mei, Huawei Yang

**Affiliations:** 1 The First Department of Breast Surgery, Guangxi Medical University Cancer Hospital, Nanning, 530021 People’s Republic of China; 2 Department of Hepatobiliary Surgery, Guangxi Medical University Cancer Hospital, No.71, Hedi Road, Nanning, 530021 Guangxi People’s Republic of China

## Correction to: Cancer Cell Int (2021) 21:55 10.1186/s12935-020-01659-0

Following the publication of the original article [1], we were notified of an error in Fig. 4.

The corrected Fig. 4 can be found in this erratum.

**Fig. 4:**
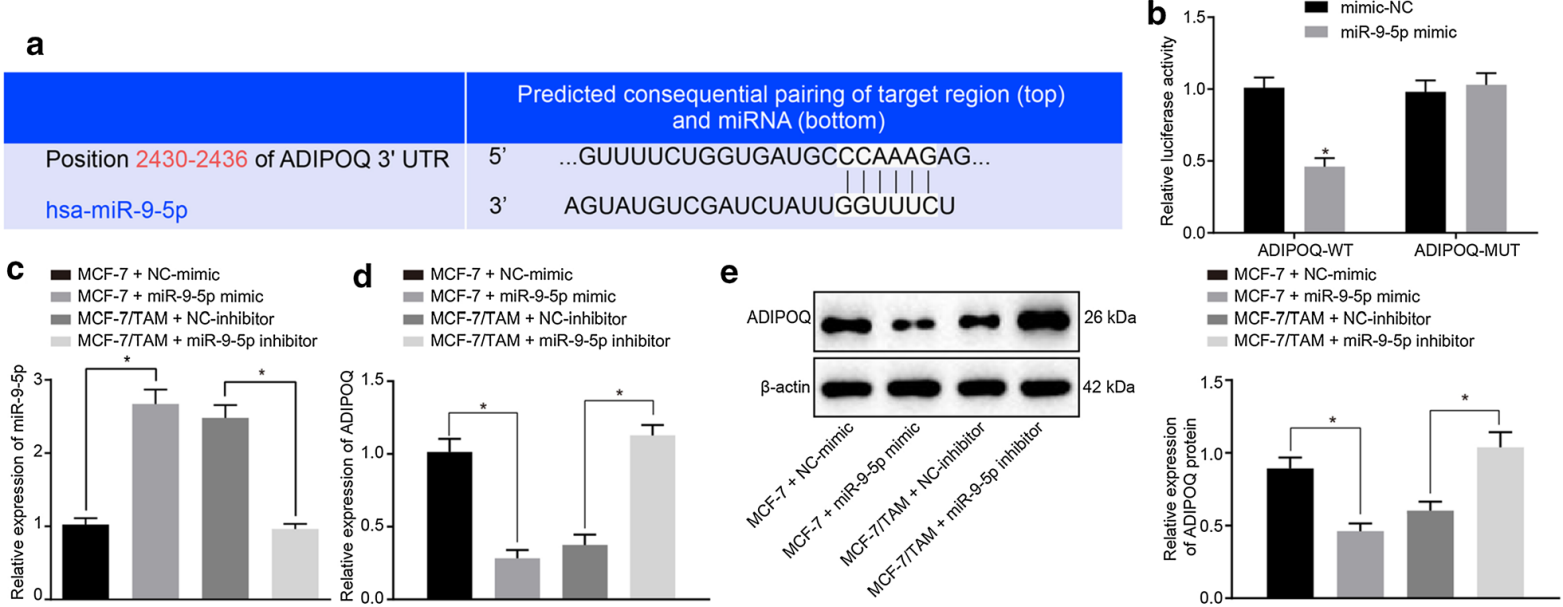
miR-9-5p targets and negatively regulates ADIPOQ. **a** The prediction of binding sites between miR-9-5p and ADIPOQ. **b** Quantitation of the luciferase activity assay. **c** The expression pattern of miR-9-5p in each group measured by RT-qPCR. **d** The mRNA expression pattern of ADIPOQ in the cells of each group measured by RT-qPCR. **e** The protein expression pattern of ADIPOQ in cells of each group evaluated by Western blot analysis. *p < 0.05. Each experiment was conducted three times independently

Errors have subsequently been identified in the original publication, and the following correction should be noted:

While analyzing the results of “nanoparticles in the concentration and size of exosomes” in the research, the result analysis of the original Fig. 1b is inaccurate. The corrected Fig. 1 is given below.

**Fig. 1:**
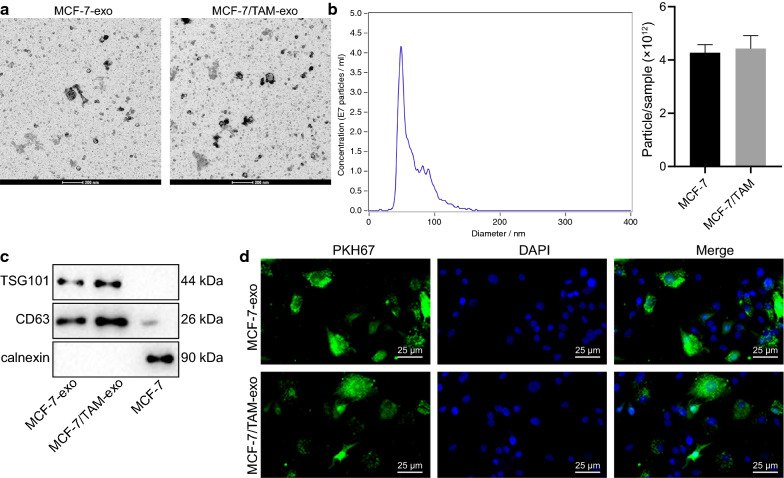
Exosomes from MCF-7/TAM cells can be transferred into the parental MCF-7 cells. **a** TEM observation of exosomes (scale bar: 200 nm). **b** Nanoparticle tracking analysis of exosome concentration and size. **c** The expression patterns of marker proteins (CD63 and TSG101) of exosomes in MCF-7-exo and MCF-7/TAM-exo detected by Western blot analysis (normalized to β-actin). **d** MCF-7-exo and MCF-7/TAM-exo (labeled with PKH67 dye) could be internationalized by parental MCF-7 cells (scale bar: 25 μm)
